# Comparative genomics of *Nocardia tsunamiensis* IFM 10818, a new source of the antibacterial macrolide nargenicin A1

**DOI:** 10.1128/spectrum.01220-25

**Published:** 2025-10-27

**Authors:** Yu Lu, Yosuke Seto, Yasumasa Hara, Akiko Takaya, Momotaka Uchida, Yoko Kusuya, Masami Ishibashi, Yuumi Nakamura, Hiroki Takahashi

**Affiliations:** 1 Graduate School of Medical and Pharmaceutical Sciences, Chiba University 12737, Chiba, Japan; 2 Faculty of Agriculture, Kagawa University 12850, Miki, Kagawa, Japan; 3 Graduate School of Pharmaceutical Sciences, Chiba University 12737, Chiba, Japan; 4 Medical Mycology Research Center, Chiba University 12737, Chiba, Japan; 5 Plant Molecular Science Center, Chiba University 12737, Chiba, Japan; 6 Biological Resource Center, National Institute of Technology and Evaluation 13594, Kisarazu, Japan; 7 Cutaneous Allergy and Host Defense, Immunology Frontier Research Center, The University of Osaka 13013, Osaka, Japan; 8 Department of Dermatology, Graduate School of Medicine, The University of Osaka 13013, Osaka, Japan; 9 Division of Microbiology and Immunology, Center for Infectious Disease Education and Research, The University of Osaka 643214, Osaka, Japan; 10 Faculty of Science, Chiba University 12737, Chiba, Japan; 11 Molecular Chirality Research Center, Chiba University 12737, Chiba, Japan; University of Melbourne, Melbourne, Australia

**Keywords:** *Nocardia*, macrolides, actinomycetes, genome analysis, genome organization, antibiotics

## Abstract

Rare actinomycetes belonging to the genus *Nocardia* are opportunistic pathogens responsible for nocardiosis, especially in immunocompromised individuals. Despite their pathogenicity, they possess diverse metabolic pathways and therefore are potential sources of novel bioactive secondary metabolites, with implications for drug discovery. However, the biosynthetic potential of many *Nocardia* strains has not been determined. In this study, we evaluated the antibiotic production abilities of 119 *Nocardia* strains through co-culturing with *Staphylococcus aureus*. Among the tested strains, *Nocardia tsunamiensis* IFM 10818 exhibited strong inhibitory activity against *S. aureus*, indicating the presence of antibacterial compounds. Subsequent chemical analysis revealed that this strain produces nargenicin A1, a macrolide antibiotic previously identified in other *Nocardia* strains. To better understand the biosynthetic potential of *N. tsunamiensis* IFM 10818, we performed whole-genome sequencing. Comparative genomic analysis revealed its phylogenetic placement within the genus *Nocardia*, demonstrating its close relationship with *Nocardia crassostreae*. Importantly, we assessed the prevalence of the biosynthetic gene cluster (BGC) responsible for nargenicin A1 production within the genus. These findings confirm that *N. tsunamiensis* IFM 10818 is a previously unreported producer of nargenicin A1. This study highlights the utility of genome mining and functional screening in discovering bioactive compounds from underexplored microbial taxa. Further exploration of *Nocardia* genomes may reveal additional cryptic BGCs with pharmaceutical potential.

**IMPORTANCE:**

The macrolide nargenicin A1, produced by certain *Nocardia* strains, exhibits a range of biological activities, most notably strong antibacterial effects against methicillin-resistant *Staphylococcus aureus*. The identification of a high-yielding producer strain is essential to support future pharmaceutical development and commercial production. In this study, we identified *Nocardia tsunamiensis* IFM 10818 as a prolific producer of nargenicin A1. This strain exhibited significant inhibitory activity against *S. aureus* in co-culture assays, and a chemical analysis confirmed the production of nargenicin A1. Genome sequencing of *N. tsunamiensis* IFM 10818 allowed for the identification of the biosynthetic gene cluster responsible for nargenicin biosynthesis, reinforcing the potential for industrial applications. These findings highlight the promise of *Nocardia* species as untapped sources of valuable secondary metabolites and emphasize the importance of strain screening and genomic analyses in the discovery and development of new antibiotics.

## INTRODUCTION

Rare actinomycetes—those belonging to genera other than *Streptomyces*—are considered highly promising resource pools for the discovery of novel antibiotics (1, 2). Among these, the gram-positive genus *Nocardia* is a ubiquitous group of environmental bacteria, best known as the causative agent of nocardiosis, an opportunistic infection that primarily affects immunocompromised individuals (3). Currently, 109 *Nocardia* species have been validly named, with approximately half considered clinically relevant (4). This number continues to grow owing to ongoing taxonomic revisions and the discovery of new species, such as *Nocardia sputorum* and *Nocardia implantans* (5, 6).

*Nocardia* species exhibit a wide range of biological activities and possess unique metabolic pathways (7). Since the discovery of nocardicin A and B from *Nocardia uniformis* var. *tsuyamanensis* ATCC 21806 (8), a wide variety of structurally diverse metabolites have been isolated from *Nocardia* strains, including macrolides (9–15). Genome mining studies have further highlighted the biosynthetic potential of *Nocardia*. For example, the genome of *N. farcinica* IFM 10152 contains 14 nonribosomal peptide synthetase (NRPS) genes (16). Komaki et al. (17) identified a diverse repertoire of NRPS and type I polyketide synthase (PKS-I) gene clusters in *Nocardia* strains, with numbers comparable to those found in *Streptomyces*. Additional genome mining has also been conducted for type II PKS clusters (18), and a comparative genomic analysis by Männle et al. (19) revealed 11 distinct PKS subfamilies and identified several novel compounds .

In this study, we screened 119 *Nocardia* strains for antibiotic production by co-culture with *Staphylococcus aureus. Nocardia tsunamiensis* IFM 10818 effectively inhibited *S. aureus* growth, and a chemical analysis identified the active compound as nargenicin A1. Whole-genome sequencing and phylogenetic analyses were performed. Furthermore, we identified the biosynthetic gene cluster (BGC) responsible for nargenicin A1 production and compared the prevalence of BGCs within the genus *Nocardia*. These findings indicate that *N. tsunamiensis* IFM 10818 is a prolific producer of nargenicin A1 and expand our understanding of the metabolic potential of *Nocardia*.

## RESULTS

### *Nocardia tsunamiensis* IFM 10818 produced nargenicin A1

To explore the potential for novel antibiotic production, we conducted a comprehensive screening of 119 *Nocardia* strains representing 89 validly named species. As a model indicator organism for antibacterial activity, we employed *S. aureus* SA113, a clinically relevant strain with a well-characterized susceptibility profile.

**Fig 1:**
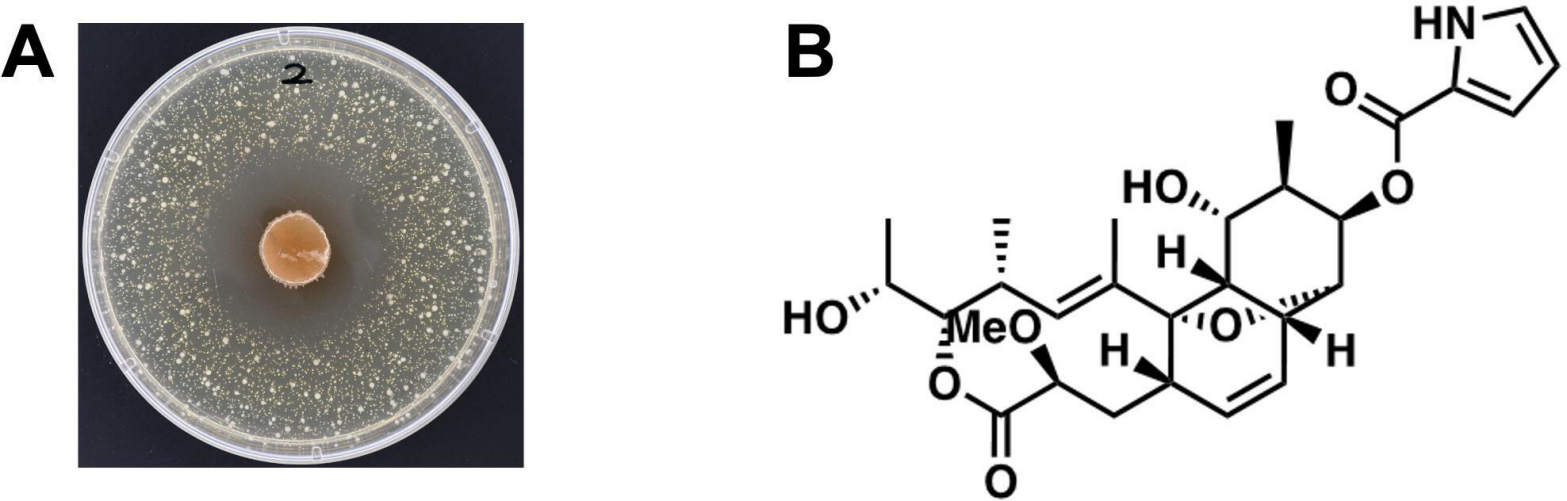
(**A**) Co-culture of *Nocardia tsunamiensis* IFM 10818 and *Staphylococcus aureus. N. tsunamiensis* IFM 10818 was first cultured on TSA at 30°C for 7 days. A 1 cm^2^ agar plug from the *N. tsunamiensis* culture was then placed at the center of LB plate inoculated with *S. aureus*. Two organisms were subsequently co-cultured on LB at 30°C for an additional 7 days. (**B**) Chemical structure of nargenicin A1.

Each *Nocardia* strain was cultured under standardized conditions and subsequently co-inoculated with *S. aureus* SA113 to assess antibacterial activity through direct growth inhibition (Table S1). Among the strains tested, *N. tsunamiensis* IFM 10818 (=DSM 44996 = OFN 05.31) (20) displayed a marked zone of inhibition, indicating potent antibacterial activity against *S. aureus* (Fig. 1A). Nine other strains, *N. brasiliensis* (IFM 0082, IFM 0235, IFM 0326, and IFM 10706), *N. italica* IFM 0852, *N. pseudobrasiliensis* IFM 12165, *N. terpenica* (IFM 0406 and IFM 0555), *N. vulneris* IFM 11698, also exhibited antibacterial activity.

To identify the bioactive compound responsible for this antibacterial effect, we performed a chemical analysis of the culture supernatant of *N. tsunamiensis* IFM 10818 (Fig. S1). NMR and mass spectrometry (MS) revealed that the compound is nargenicin A1, a macrolide with established activity against gram-positive pathogens (Fig. 1B; Fig. S2 and S3; Table S2). These results highlight *N. tsunamiensis* IFM 10818 as a prolific producer of nargenicin A1.

Nargenicin A1, originally designated as CP-47444, was first reported in 1977 as an antibiotic compound isolated from cultures of *N. argentinensis* (21, 22). Since then, its production has been experimentally confirmed in *Nocardia* sp. CS682 and *N. arthritidis* AUSMDU00012717 (23, 24). Based on our findings, *N. tsunamiensis* IFM 10818 represents the fourth known *Nocardia* strain capable of producing nargenicin A1 (Table 1).

**TABLE 1:** *Nocardia* strains producing nargenicin A1

Species	Strain ID	Country	Date of isolation	Source	Refs.
*Nocardia tsunamiensis*	IFM 10818 = DSM 44996 = OFN 05.31	Antibes, France	January 2004	Clinical (nocardiosis)	This study Blanc et al. (20)
*Nocardia argentinensis*	ATCC 31306	Argentina		Soil	Celmer et al. (21)
*Nocardia* sp.	CS682	Jeonnam, Korea		Soil	Sohng et al. (23)
*Nocardia arthritidis*	AUSMDU00012717	Australia	2011	Clinical (sputum)	Pidot et al. (24)

To assess the antibacterial spectrum of nargenicin A1, we performed minimum inhibitory concentration (MIC) assays against a panel of both gram-positive and gram-negative bacterial strains. This panel included *S. aureus* (including methicillin-resistant *S. aureus*, MRSA), *Bacillus subtilis* 168, *Salmonella enterica* serovar Typhimurium χ3306, and *Escherichia coli*. Consistent with previous studies (18, 25), nargenicin A1 showed potent inhibitory activity against *S. aureus*, including MRSA strains, with efficacy comparable to that of vancomycin (Table 2). While no significant inhibitory effect was observed against *B. subtilis* 168, *S.* Typhimurium χ3306, and wild-type *E. coli* strains, nargenicin A1 exhibited antibacterial activity against efflux pump-deficient strains, such as *E. coli* Δ*acrAB* and *S*. Typhimurium Δ*tolC* (26, 27). These results suggest that nargenicin A1 has a relatively narrow antibacterial spectrum primarily targeting *S. aureus*, while its activity against gram-negative bacteria is limited and likely hindered by intrinsic resistance mechanisms, such as efflux pumps.

**TABLE 2:** Antibiotic activity of nargenicin A1

Species	Strain	MIC (mg/L)			
		Vancomycin		Nargenicin A1	
		#1	#2	#1	#2
*Staphylococcus aureus*	ATTC 29213	0.5	0.5	0.25	0.5
*Staphylococcus aureus* (MSSA)^*a*^	2021-I1397	1	1	0.25	0.25
*Staphylococcus aureus* (MRSA)^*b*^	2021-I0946	1	1	0.25	0.25
*Staphylococcus aureus* (LVFX^R^)^*c*^	2022-I0071	0.5	0.5	0.25	0.25
*Bacillus subtilis*	168	0.25	0.25	>128	128
*Escherichia coli*	ATCC 25922	>128	>128	>128	>128
*Escherichia coli*	CSH2	128	128	>128	>128
*Escherichia coli* Δ*acrAB*	KAM3	>128	>128	1	1
*Salmonella* Typhimurium WT	χ3306	>128	>128	>128	>128
*Salmonella* Typhimurium Δ*tolC*	CS10325	>128	>128	1	1

^*a*^ MSSA refers to a methicillin-susceptible *Staphylococcus aureus*.

^*b*^ MRSA refers to methicillin-resistant *Staphylococcus aureus*.

^*c*^ LVFX^R^ refers to levofloxacin-resistant.

### Genome sequence of *N. tsunamiensis* IFM 10818

To elucidate the BGC responsible for nargenicin A1 production (hereafter referred to as the *ngn* BGC) in *N. tsunamiensis* IFM 10818, we performed whole-genome sequencing using a hybrid approach combining long-read Oxford Nanopore sequencing with short-read Illumina sequencing. The assembled genome of *N. tsunamiensis* IFM 10818 included a chromosome and four plasmids, pNT1, pNT2, pNT3, and pNT4 (Table 3; Fig. S4), with total lengths of 8,413,449, 170,972, 93,799, 36,741, and 25,384 bp, respectively. In total, 8,078 protein-coding genes were predicted across the entire genome.

**TABLE 3:** Genome information for *Nocardia tsunamiensis* IFM 10818

	Chromosome	pNT1	pNT2	pNT3	pNT4
Topology	Circular	Circular	Circular	Circular	Circular
Length (bp)	8,413,449	170,972	93,799	36,741	25,384
Copy no.^*a*^	1	1.4	1.8	2.5	3.4
GC%	68.2	69.0	67.3	67.3	68.1
# of protein-coding genes	7,690	197	120	40	31
# of rRNA	12	0	0	0	0
# of tRNA	72	0	0	0	0

^*a*^ The copy numbers of plasmids were estimated using CoverM.

**Fig 2:**
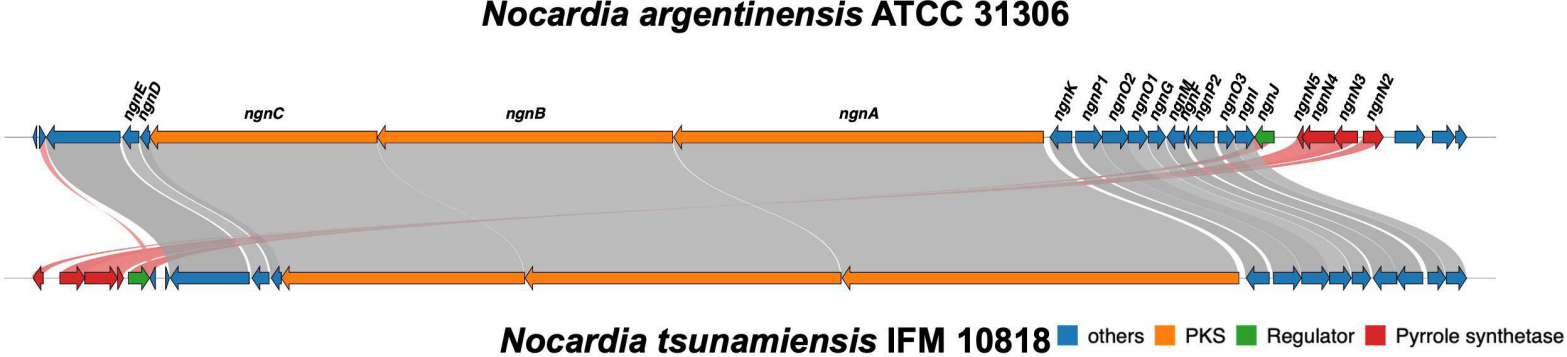
Alignment of the *ngn* biosynthetic gene cluster (BGC) between *Nocardia argentinensis* ATCC 31306 and *Nocardia tsunamiensis* IFM 10818. The organization of BGCs of both strains were compared.

To explore the secondary metabolite potential of this strain, we utilized antiSMASH for BGC prediction. We identified 32 putative BGCs within the genome, all of which were located on the chromosome (Table S3). The *ngn* BGC was annotated as BGC11 (94,529 nt). A comparative analysis revealed that the *ngn* BGC in *N. tsunamiensis* IFM 10818 shares a high degree of synteny with the well-characterized *ngn* BGC from *N. argentinensis* ATCC 31306 (28), a known producer of nargenicin A1. However, noteworthy differences in gene organization were observed between the two strains. In particular, there were differences in the positions of *ngnN2*, *ngnN3*, *ngnN4*, *ngnN5*, and *ngnJ*. In *N. argentinensis*, these genes were clustered adjacent to *ngnI*, whereas in *N. tsunamiensis*, they were situated in proximity to a different open reading frame, designated ORF6 (Fig. 2). This genomic transposition may reflect evolutionary divergence in gene regulation or cluster organization and could potentially influence the biosynthesis or structure of nargenicin A1.

### Phylogenetic position of *N. tsunamiensis* IFM 10818

**Fig 3:**
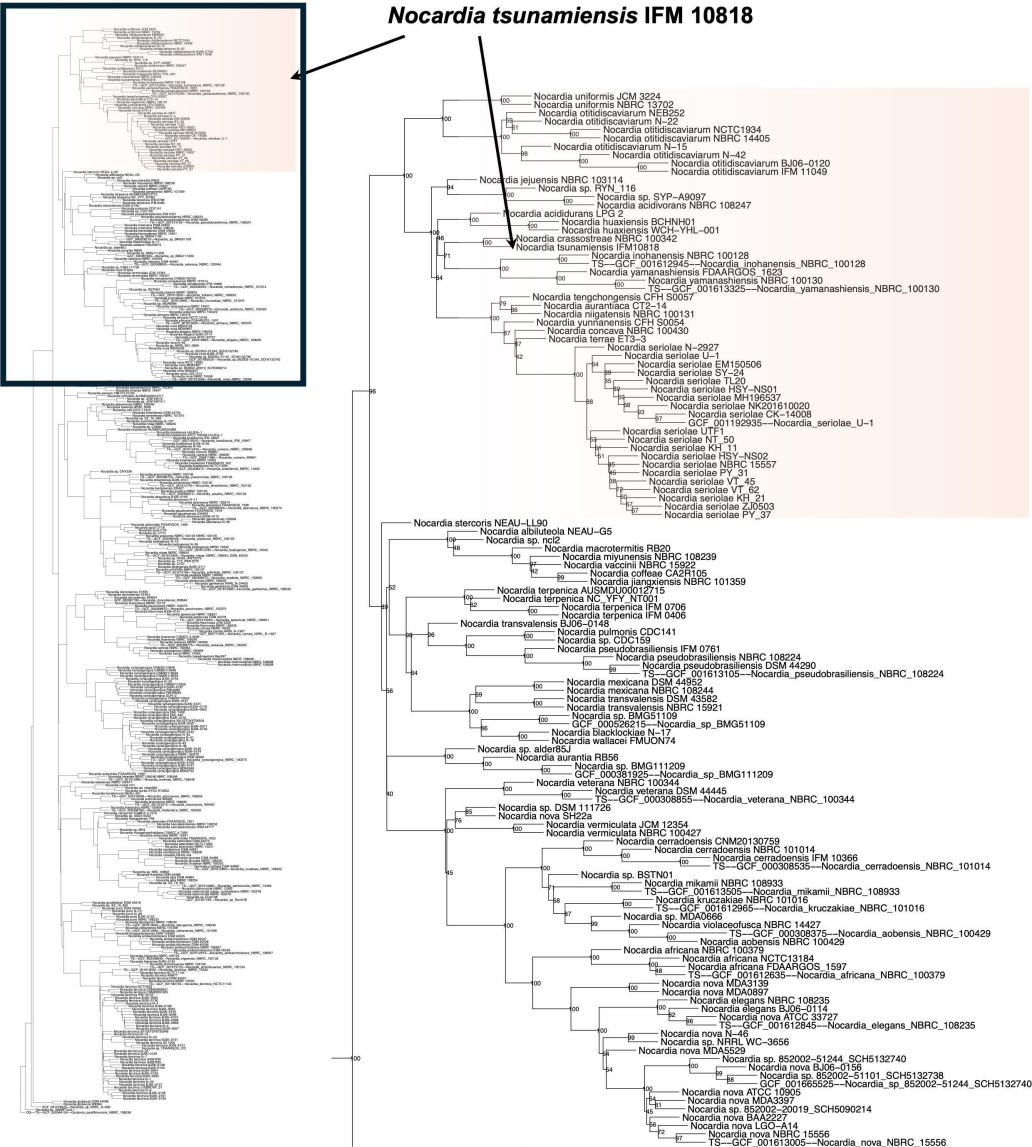
Phylogenetic position of *Nocardia tsunamiensis* IFM 10818 within the genus *Nocardia*. The maximum likelihood phylogenetic tree with the GTR model was constructed based on 10 MLST genes selected using the autoMLST pipeline. The tree highlights the relationship between *N. tsunamiensis* IFM 10818 and other *Nocardia* species.

To determine the position of *N. tsunamiensis* IFM 10818 within the genus, we conducted a comprehensive phylogenetic analysis based on whole-genome sequences from 337 *Nocardia* strains (Fig. 3; Tables S4 and S5). *N. tsunamiensis* IFM 10818 was most closely related to *Nocardia crassostreae* NBRC 100342.

We also investigated the distribution of the *ngn* BGC across the *Nocardia* genus. Using comparative genome analysis and cluster alignment tools, we identified the *ngn* BGC in five *Nocardia* strains: *N. tsunamiensis* IFM 10818, *Nocardia* sp. CS682, *N. arthritidis* AUSMDU00012717, *N. transvalensis* BJ06-0148, and *N. otitidiscaviarum* NEB252 (Table 1; Fig. S5). Notably, all five *ngn* BGCs showed a high degree of sequence similarity and conserved gene organization, indicating that the core structure of this gene cluster is well preserved across these species (Fig. S6; Table S6).

### Pan-genome analysis of BGC conservation across *Nocardia* species

**Fig 4:**
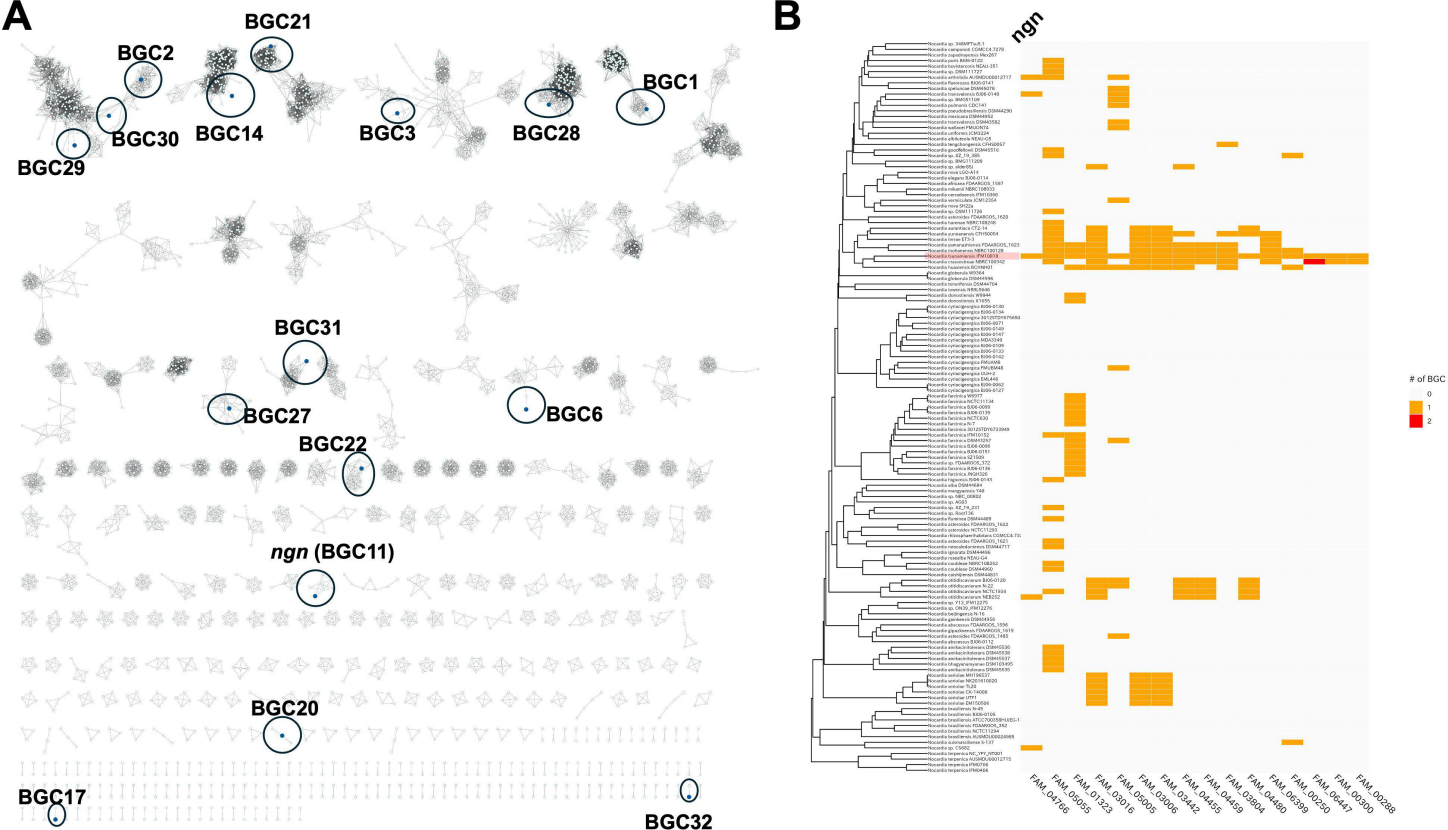
Pan-genome analysis of *Nocardia* biosynthetic gene clusters (BGCs). (**A**) Overview of the BGC network. The BGCs in *N. tsunamiensis* IFM 10818 are indicated. (**B**) Distribution of 16 BGCs in *N. tsunamiensis* IFM 10818. Note that *N. tsunamiensis* IFM 10818 is highlighted in red.

To investigate the conservation of the *ngn* BGC, a pan-genome BGC analysis was performed using 131 *Nocardia* genomes (Table S7). In total, 3,962 predicted BGCs were identified across these genomes (Table S8). These BGCs were assigned to 1,456 gene cluster families (GCFs) using the Big-Scape pipeline (Fig. 4A; Fig. S7). Of 32 BGCs present in *N. tsunamiensis* IFM 10818, 16 were unique to this strain, while the remaining 16 were conserved among *Nocardia* species (Fig. 4B). Notably, 11 of the 16 BGCs in *N. tsunamiensis* IFM 10818 were also detected in *N. crassostreae* NBRC 100342 (Fig. 4B; Table S9), indicating a close relationship between the two strains. Furthermore, three specific BGCs, that is, BGC17 (NRPS; FAM_00300), BGC20 (Others; FAM_06447), and BGC32 (PKSI; FAM_00288), were found exclusively in *N. tsunamiensis* IFM 10818 and *N. crassostreae* NBRC 100342 (Fig. 4B; Fig. S8).

These findings provide phylogenetic insight into *N. tsunamiensis* IFM 10818 and expand our understanding of the distribution and evolution of the *ngn* BGC within the genus. The results underscore the broader potential of *Nocardia* species as untapped resources for antibiotic discovery.

## DISCUSSION

We identified *N. tsunamiensis* IFM 10818 as a prolific producer of the macrolide antibiotic nargenicin A1 through the screening of 119 *Nocardia* strains (Table S1). This strain represents the fourth known nargenicin A1 producer, following *N. argentinensis* ATCC 31306, *Nocardia* sp. CS682, and *N. arthritidis* AUSMDU00012717 (21, 23, 24). Nargenicin A1 is an ether-bridged macrolide with potent antimicrobial activity, particularly against *S. aureus* and *Micrococcus luteus* (22).

The clinical relevance of this compound is underscored by its effectiveness against MRSA, a major global health threat due to its high pathogenicity and frequent involvement in nosocomial outbreaks, including those occurring in neonatal intensive care units (29, 30). Mechanistically, nargenicin A1 exerts antibacterial activity by binding to DnaE, a DNA polymerase III α subunit, thereby disrupting DNA synthesis and effectively inhibiting the growth of MRSA (25). Beyond its antibacterial properties, nargenicin A1 exhibits multiple biological activities; for example, it promotes the differentiation of acute myeloid leukemia cells, exhibits anti-inflammatory effects, and protects cells against oxidative stress (31–34). This diverse range of bioactivities supports the value of nargenicin A1 as a drug-like molecule with potential therapeutic applications beyond antibacterial use. Accordingly, developing methods to enhance the yield of nargenicin A1 is of significant interest for pharmaceutical applications. Previous studies have demonstrated that metabolic engineering strategies can effectively boost its production (35, 36).

Our study demonstrated that nargenicin A1 production in *N. tsunamiensis* IFM 10818 could be reliably induced under laboratory conditions, suggesting that this strain is a promising biological resource for the scalable production of nargenicin A1. Moreover, the gene organization of the *ngn* BGC differs between *N. tsunamiensis* IFM 10818 and *N. argentinensis* ATCC 31306 (Fig. 2; Fig. S5), and such differences may affect the efficacy of nargenicin A1 production. Collectively, these findings contribute to the growing list of *Nocardia* strains with high secondary metabolite biosynthetic potential.

Interestingly, *N. tsunamiensis* IFM 10818 was originally isolated from a patient who survived the 2004 tsunami in Thailand (20). A phylogenomic analysis revealed that this strain is most closely related to *N. crassostreae* NBRC 100342, a species isolated from oysters (Fig. 3) (37). This close relationship suggests that the two species share a common marine or estuarine ecological niche. Supporting this idea, genome mining revealed three BGCs shared exclusively between *N. tsunamiensis* IFM 10818 and *N. crassostreae* NBRC 100342 (Fig. 4B; Fig. S8). These findings imply that marine-derived *Nocardia* strains could be rich and underexplored sources of novel bioactive secondary metabolites.

Finally, our comparative genomic analysis revealed that the *ngn* BGC is present in two additional *Nocardia* strains—*N*. *transvalensis* BJ06-0148 and *N. otitidiscaviarum* NEB252 (Fig. S5 and S6; Table S6). Although the production of nargenicin A1 in these strains has not yet been experimentally validated, the conservation of the *ngn* BGC suggests their potential to synthesize this compound. Notably, the *ngn* BGC was detected in only one out of eight *N. otitidiscaviarum* strains and one out of three *N. arthritidis* strains examined. Its scattered distribution among taxa (Fig. 4B) suggests that the *ngn* BGC could be strain-specific rather than species-wide. This strain-level specificity underscores the importance of genomic screening across diverse isolates for natural product discovery.

Taken together, our findings establish *N. tsunamiensis* IFM 10818 as a new and valuable producer of nargenicin A1, expand the known diversity of *Nocardia* strains harboring the *ngn* BGC, and suggest that further exploration of marine-associated *Nocardia* species may yield novel bioactive compounds with therapeutic potential.

## MATERIALS AND METHODS

### Strains and culture conditions

All *Nocardia* strains used in this study were provided by the Medical Mycology Research Center, Chiba University with support in part by National BioResource Project (NBRP), MEXT, Japan (https://nbrp.jp/) (Table S1). The *Nocardia* strains were pre-cultured on trypticase soy agar (TSA: BD, Franklin Lakes, NJ, USA) at 30 °C for 7 days. *N. tsunamiensis* IFM 10818 was inoculated into 5 mL of trypticase soy broth (TSB; BD) supplemented with glass beads and incubated at 30 °C with shaking at 160 rpm for 7 days. *S. aureus* SA113 was used as the target bacterium.

### Antibacterial activity screening

Antimicrobial activity screening of *Nocardia* strains was performed using a co-culture method. Each *Nocardia* strain was cultured on TSA for 7 days at 30 °C. Subsequently, a 1 cm^2^ agar plug containing the *Nocardia* culture was placed onto an LB plate with *S. aureus* SA113 and incubated for 1 day at 30 °C. *S. aureus* SA113 was pre-cultured and mixed into the LB plate after autoclaving, prior to solidification. Antibacterial activity was assessed based on growth inhibition for screening purposes.

### Identification of antibacterial compounds

A 10 L culture of *N. tsunamiensis* IFM 10818 was centrifuged at 4,720 × *g* for 20 min to separate the supernatant and mycelial biomass. The supernatant was thoroughly extracted with an equal volume of ethyl acetate (EtOAc). After the extraction, the organic solvent was evaporated and dried under reduced pressure. As a result, 1.37 g of EtOAc extract was obtained. The EtOAc extract was dissolved in methanol (MeOH) and subjected to octadecylsilyl (ODS) column chromatography using Chromatorex ODS (*ϕ* 25 × 200 mm; Fuji Silysia, Aichi, Japan) with a stepwise MeOH–H_2_O gradient. Seven fractions (1A–1G) were collected (Fig. S1). The antimicrobial activity of each fraction was evaluated using the disk diffusion method. Sterile 8-mm-diameter disks were placed on LB agar plates seeded with *S. aureus* at a final concentration of 8.4 × 10^5^ cells/mL. Fraction 1D (MeOH:H_2_O = 8:2, 37.2 mg), which exhibited antibacterial activity, was further purified by reversed-phase HPLC (COSMOSIL 5 _C18_ AR-II column, *ϕ* 10.0 × 250 mm; Nacalai Tesque, Kyoto, Japan) using 60% MeOH as the eluent at a flow rate of 4 mL/min. This yielded compound 1 (fraction 2B, 13.8 mg, retention time = 15.5 min). Compound 1 was identified as nargenicin A1 based on comparisons of its NMR (24) and mass spectral data with those previously reported in the literature (21). The ^1^H and ^13^C NMR spectral data are presented in Fig. S2 and Table S2. The ESIMS spectrum of nargenicin A1 showed an [M + H]^+^ ion at *m/z* 516 (Fig. S3).

### Antibiotic susceptibility testing

MIC assays were performed in duplicate according to CLSI methods (38). *S. aureus* strains, including *S. aureus* (including MRSA), *B. subtilis* 168, wild-type and TolC-deficient (CS10325) strains of *S.* Typhimurium χ3306 (39), and *E. coli* strains, were utilized to test the antibacterial activity of purified nargenicin A1. *S. aureus* ATCC 29213 and *E. coli* ATCC 25922 strains were used as quality control strains.

### Genome sequencing

Genomic DNA from *N. tsunamiensis* IFM 10818 was extracted using a combination of the phenol–chloroform method and the Genomic-Tip 20/G Kit (Qiagen, Hilden, Germany). Whole-genome sequencing was performed using a hybrid approach combining Oxford Nanopore Technologies (ONT) and Illumina NovaSeq platforms (250 bp paired-end). For ONT sequencing, a DNA library was prepared using a Short Read Eliminator Kit (Nippon Genetics, Tokyo, Japan) and Ligation Sequencing Kit (SQK-LSK110, ONT). Sequencing was conducted on a MinION device equipped with an R9.4.1 flow cell, generating a total of 22,043 reads. Basecalling was performed using Guppy v6.4.2+97a7f06, following the manufacturer’s instructions. The ONT reads were trimmed and filtered using Porechop v0.2.4 (https://github.com/rrwick/Porechop) and NanoFilt v2.8.0  (40) with the options -q 10 -l 1000 --headcrop 50. Genome assembly was carried out using Flye v2.9-b1768  (41), and error correction was performed using NextPolish v1.4.1  (42). Circularization of the genome was achieved using Circlator v1.5.5  (43), in combination with BWA v0.7.17  (44), MUMmer v3.1  (45), Prodigal v2.6.3  (46), SAMtools v1.14  (47), and SPAdes v3.15.5  (48). The circularization of plasmid pNT1 was facilitated by PCR (Table S10). Plasmid copy numbers were estimated using CoverM v0.6.1 (49). Gene annotation was performed using Prokka v1.14.6  (50) and bakta v1.11.3 (51), and BGCs were predicted using antiSMASH v8.0.1  (52).

### Phylogenetic and bioinformatic analyses

In total, 352 *Nocardia* genome sequences were downloaded from GenBank. Genome completeness was assessed using CheckM v1.1.10  (53), and 336 genomes with ≥95% completeness were retained for further analyses (Table S3). A phylogenetic analysis based on the maximum likelihood method was conducted using IQ-tree v1.6.12 (54) under the GTR model with 1,000 bootstrap replicates, as implemented in the autoMLST pipeline (55). To identify the presence of the *ngn* BGC, a BLASTp search v2.12.0+  (56) was performed using the PKS-I gene sequence from *N. argentinensis* (accession no. AXG22407.1) as the query against the protein data sets of all *Nocardia* genomes. BGC alignments and visualizations were generated using the R package geneviewer v0.1.8  (57) and Easyfig v2.2.2  (58).

An absence and presence matrix of BGCs in *Nocardia* strains was generated using BiG-SCAPE v1.1.8 (59). Initially, the BGCs for each strain were predicted using antiSMASH from 131 *Nocardia* genomes containing fewer than 51 contigs (Table S7). These predicted BGCs were subsequently clustered into GCFs using BiG-SCAPE with default parameters. The MIBiG database v3.0 (60) was included as a reference to facilitate the identification and classification of known BGCs. The network was visualized using Cytoscape v3.9.1 (61).

## Data Availability

The whole-genome sequences of *N. tsunamiensis* IFM 10818 were deposited at DDBJ/EMBL/GenBank under the accession numbers BAAHJH010000001–BAAHJH010000005. Raw reads have been deposited in the DDBJ BioProject database (BioProject accession number PRJDB20550).

## SUPPLEMENTAL MATERIAL

Figure S1 spectrum.01220-25-s0001.pdf Workflow of compound identification.

Figure S2 spectrum.01220-25-s0002.pdf NMR spectra for nargenicin A1.

Figure S3 spectrum.01220-25-s0003.pdf ESIMS spectrum for nargenicin A1.

Figure S4 spectrum.01220-25-s0004.pdf Circular maps of the genome.

Figure S5 spectrum.01220-25-s0005.pdf Phylogenetic positions of strains containing the *ngn* biosynthetic gene cluster.

Figure S6 spectrum.01220-25-s0006.pdf Sequence alignment of the *ngn* biosynthetic gene cluster.

Figure S7 spectrum.01220-25-s0007.pdf Phylogenetic relationships based on biosynthetic gene clusters and conservation of gene cluster families.

Figure S8 spectrum.01220-25-s0008.pdf Biosynthetic gene clusters present in *Nocardia tsunamiensis* IFM 10818 and *Nocardia crassostreae* NBRC 100342.

Tables S1 to S10 spectrum.01220-25-s0009.xlsx Table S1: *Nocardia* strains screened in this study. Table S2: NMR spectrum of nargenicin A1. Table S3: Thirty-two biosynthetic gene clusters in *Nocardia tsunamiensis* IFM 10818. Table S4: *Nocardia* strains used for a phylogenomic analysis. Table S5: Ten MLST genes. Table S6: The presence and absence of genes in the *ngn* biosynthetic gene cluster. Table S7: *Nocardia* strains used for a pan-BGC analysis. Table S8: Predicted biosynthetic gene clusters in 131 *Nocardia* strains. Table S9: Shared biosynthetic gene clusters in *Nocardia tsunamiensis* and *N. crassostreae*. Table S10: Primer sequences used for circularization of pNT1.

ASM does not own the copyrights to Supplemental Material that may be linked to, or accessed through, an article. The authors have granted ASM a non-exclusive, world-wide license to publish the Supplemental Material files. Please contact the corresponding author directly for reuse.
