# Supplementary material for: Comparative genomics of Nocardia tsunamiensis IFM 10818, a new source of the antibacterial macrolide nargenicin A1
Source: Microbiol Spectr. 2025 Oct 27;13(12):e01220-25. doi: 10.1128/spectrum.01220-25 (PMC12671133; doi:10.1128/spectrum.01220-25)
Supplement: Figure S1 — Workflow of compound identification. [file spectrum.01220-25-s0001.pdf]

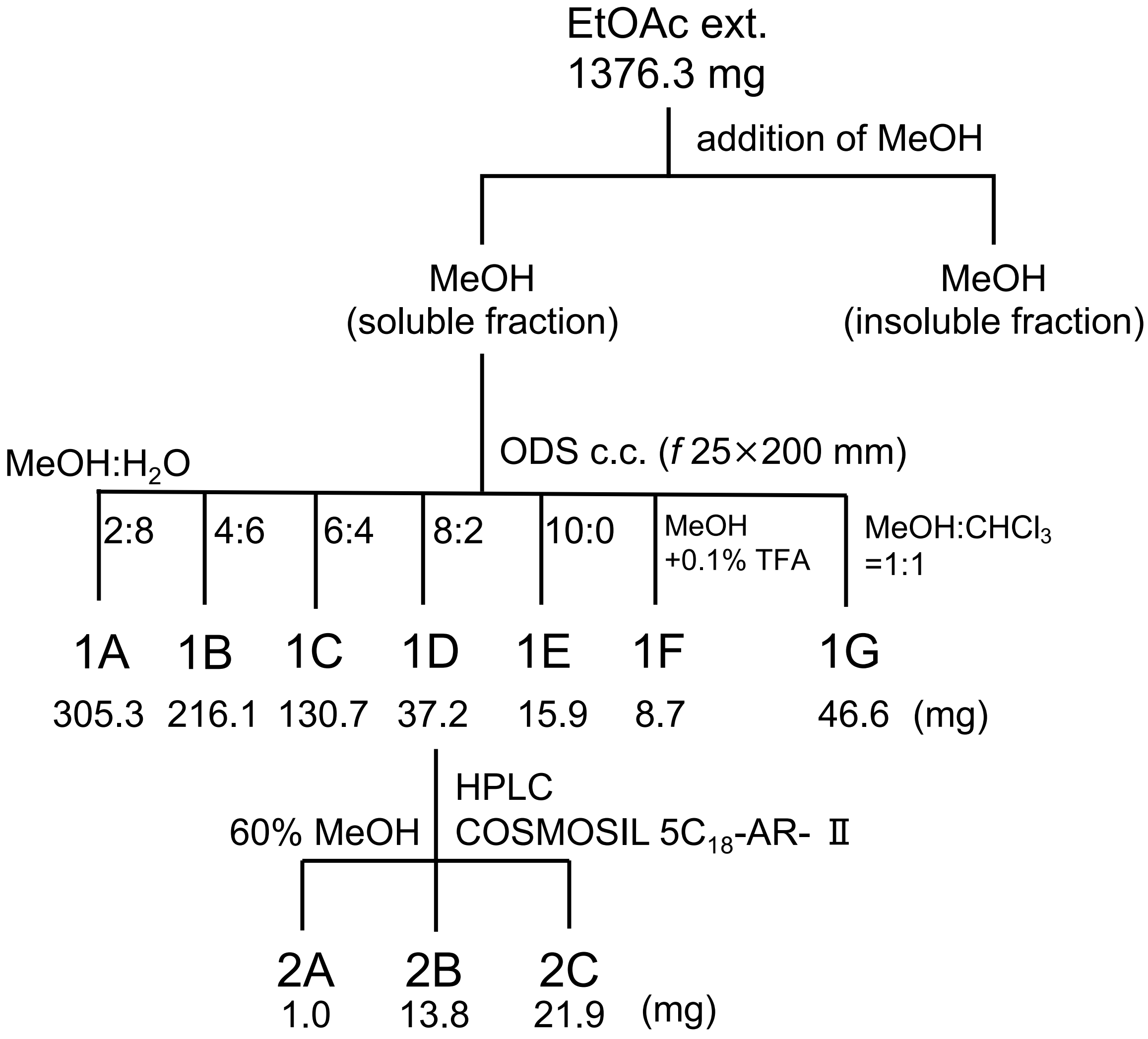

Supplementary Figure S1. Workflow of compound identification. The EtOAc extract was dissolved in MeOH, and fractions 1A–1G were obtained by HPLC. Fraction 1D, which exhibited the highest antibacterial activity, was further purified using 60% MeOH. Compound identification was then performed on fraction 2B, which showed the highest activity after this second purification step.
