# Supplementary figures and images for: Comparative genomics of Nocardia tsunamiensis IFM 10818, a new source of the antibacterial macrolide nargenicin A1
Source: Microbiol Spectr. 2025 Oct 27;13(12):e01220-25. doi: 10.1128/spectrum.01220-25 (PMC12671133; doi:10.1128/spectrum.01220-25)

**A**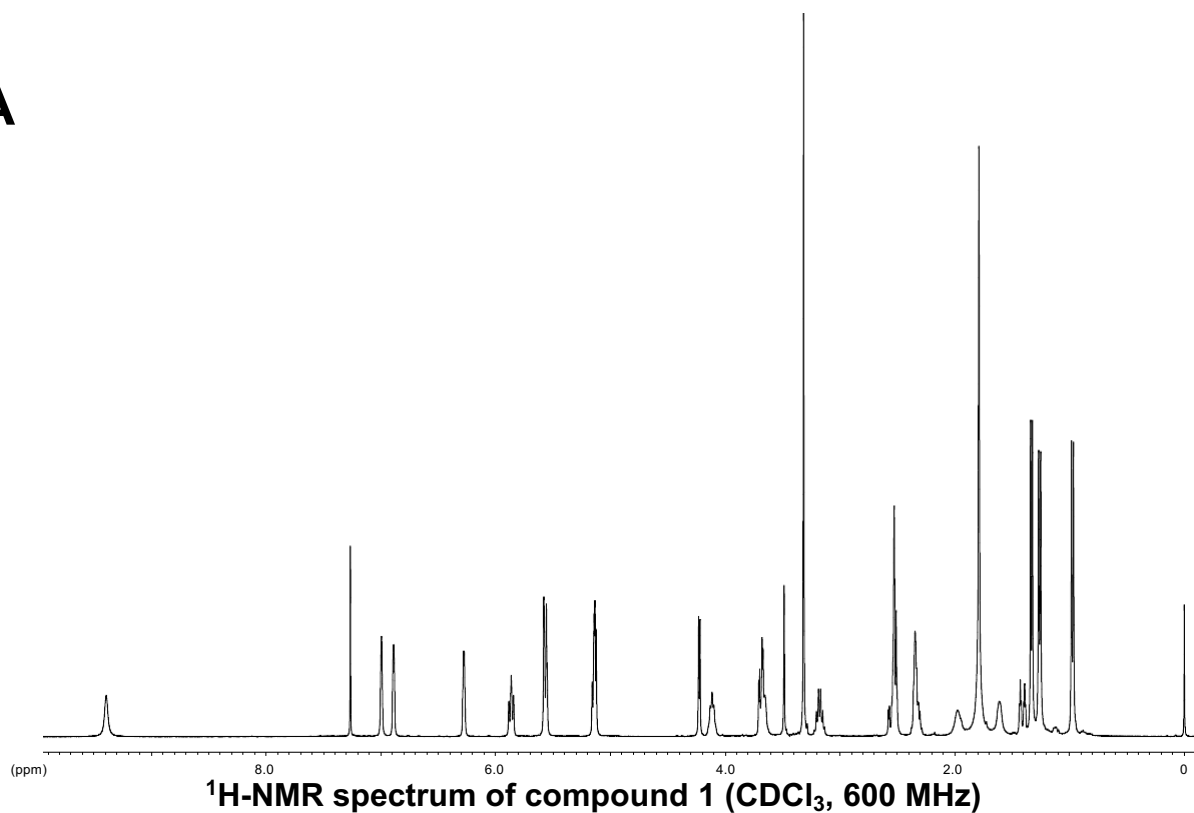**B**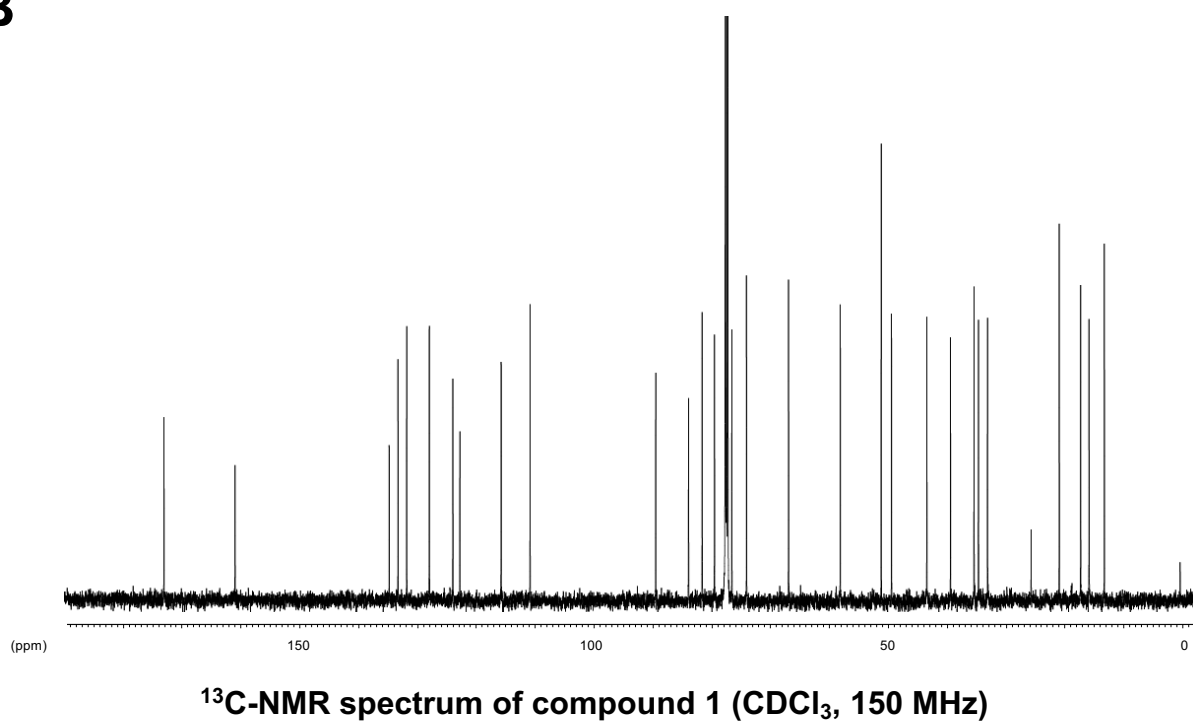

**Supplementary Figure S2.** NMR spectra for nargenicin A1. **(A)**  $^1\text{H}$  NMR spectra. **(B)**  $^{13}\text{C}$  NMR spectra.

Supplement: Figure S2 — NMR spectra for nargenicin A1. [file spectrum.01220-25-s0002.pdf]

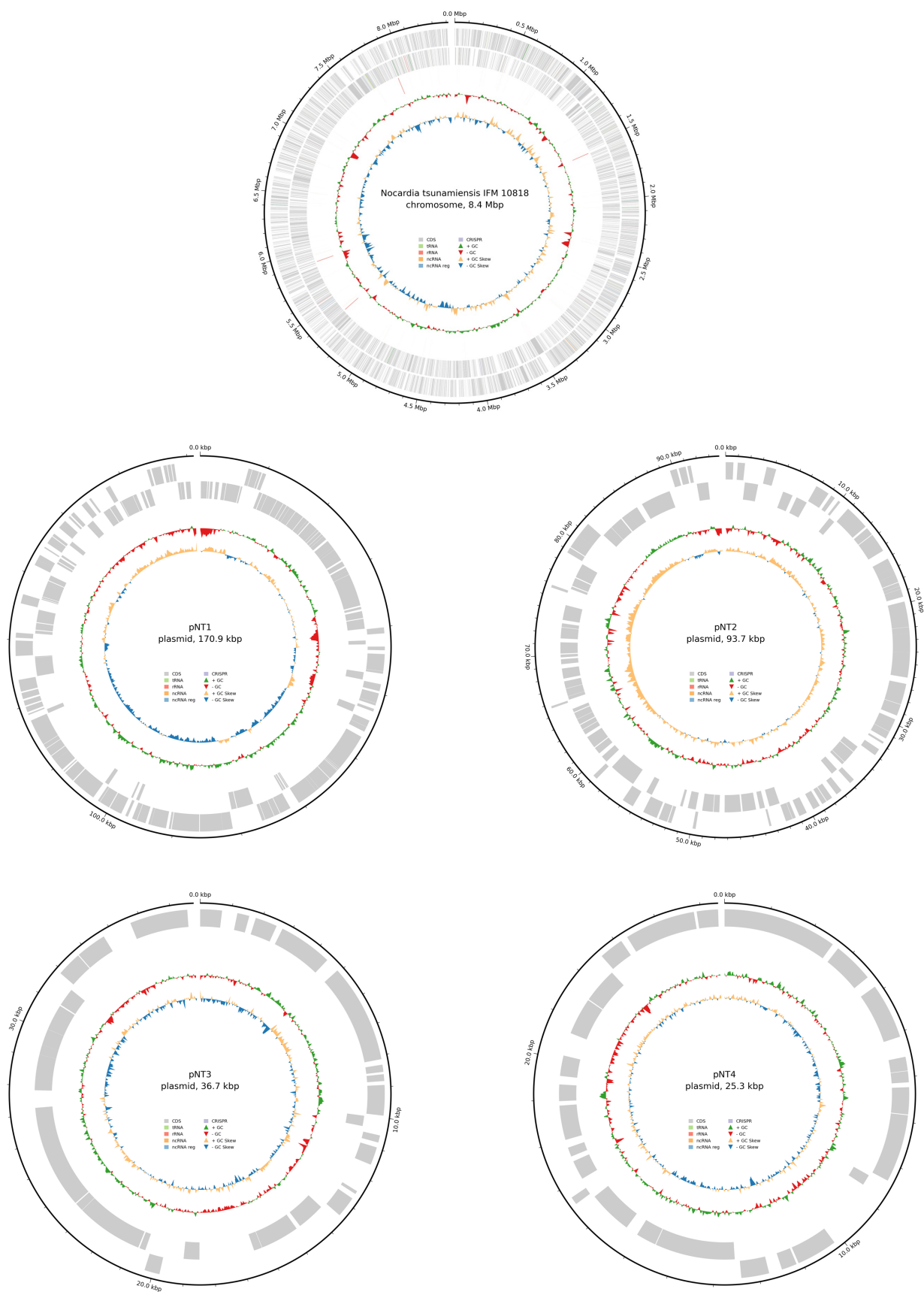

**Supplementary Figure S4.** Circular maps of the chromosome (8,413,449 bp), pNT1 (170,972 bp), pNT2 (93,799 bp), pNT3 (36,741 bp), and pNT4 (25,384 bp).

Supplement: Figure S4 — Circular maps of the genome. [file spectrum.01220-25-s0004.pdf]
