# Supplementary material for: Comparative genomics of Nocardia tsunamiensis IFM 10818, a new source of the antibacterial macrolide nargenicin A1
Source: Microbiol Spectr. 2025 Oct 27;13(12):e01220-25. doi: 10.1128/spectrum.01220-25 (PMC12671133; doi:10.1128/spectrum.01220-25)
Supplement: Figure S3 — ESIMS spectrum for nargenicin A1. [file spectrum.01220-25-s0003.pdf]

**A**

Positive ion

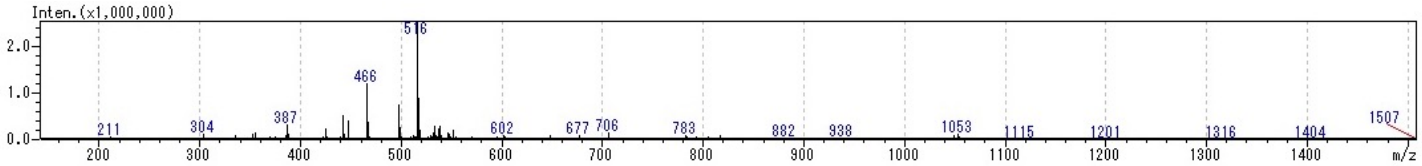


**B**

Negative ion

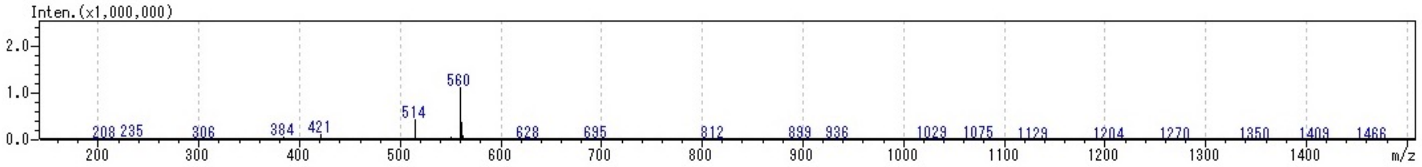


**Supplementary Figure S3.** ESIMS spectrum for nargenicin A1. **(A)** Positive ion mode. The $[M+H]^+$ ion was observed at *m/z* 516, corresponding to nargenicin A1. **(B)** Negative ion mode.
