# Supplementary material for: Comparative genomics of Nocardia tsunamiensis IFM 10818, a new source of the antibacterial macrolide nargenicin A1
Source: Microbiol Spectr. 2025 Oct 27;13(12):e01220-25. doi: 10.1128/spectrum.01220-25 (PMC12671133; doi:10.1128/spectrum.01220-25)
Supplement: Figure S5 — Phylogenetic positions of strains containing the ngn biosynthetic gene cluster. [file spectrum.01220-25-s0005.pdf]

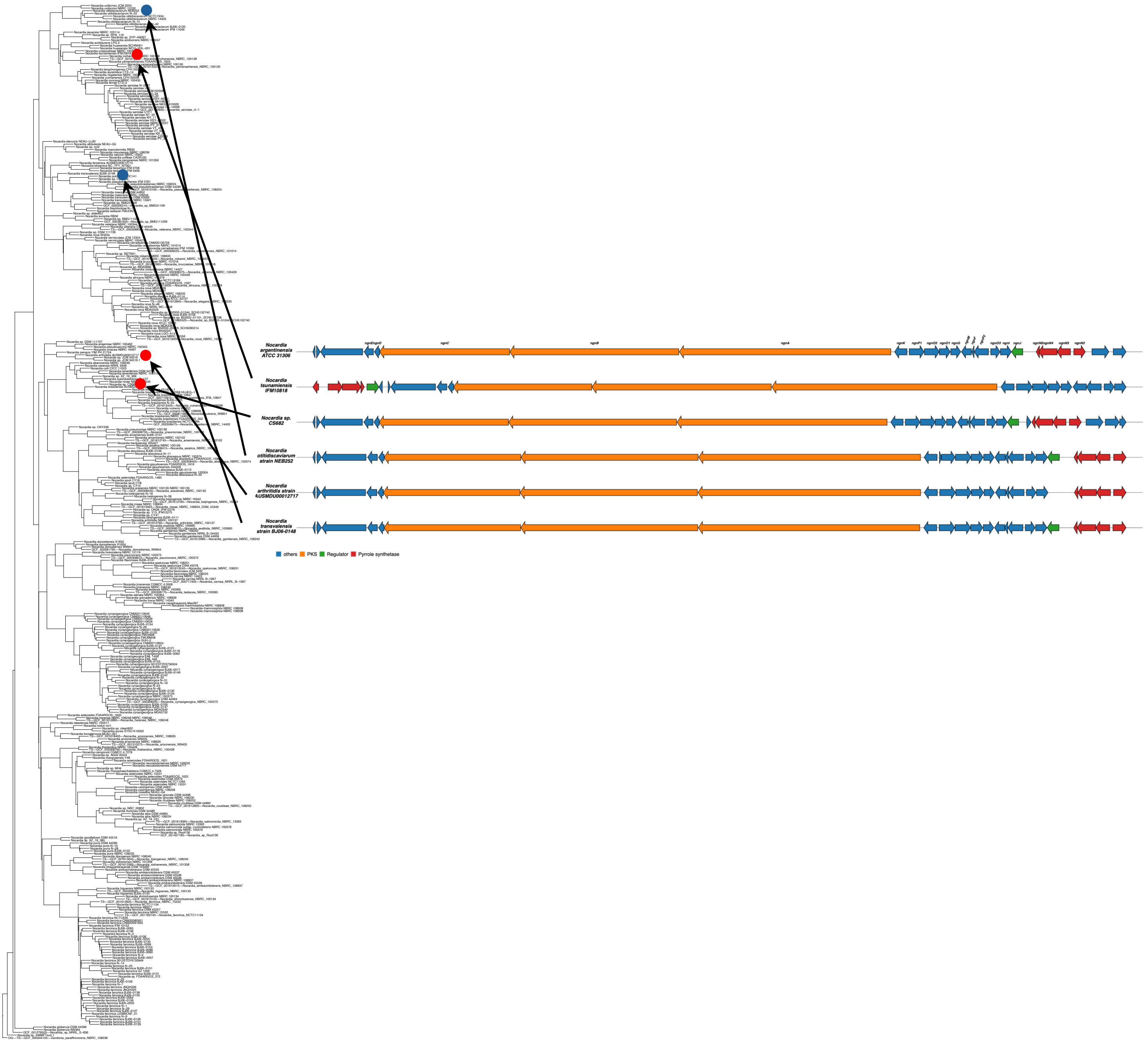

Supplementary Figure S5. Phylogenetic positions of strains containing the *ngn* biosynthetic gene cluster (BGC). The tree illustrates the evolutionary placement of strains harboring the *ngn* BGC within the genus *Nocardia*. Red and blue circles represent the strains with and without experimentally validated nargenicin A1 production, respectively.
