# Supplementary material for: Comparative genomics of Nocardia tsunamiensis IFM 10818, a new source of the antibacterial macrolide nargenicin A1
Source: Microbiol Spectr. 2025 Oct 27;13(12):e01220-25. doi: 10.1128/spectrum.01220-25 (PMC12671133; doi:10.1128/spectrum.01220-25)
Supplement: Figure S6 — Sequence alignment of the ngn biosynthetic gene cluster. [file spectrum.01220-25-s0006.pdf]

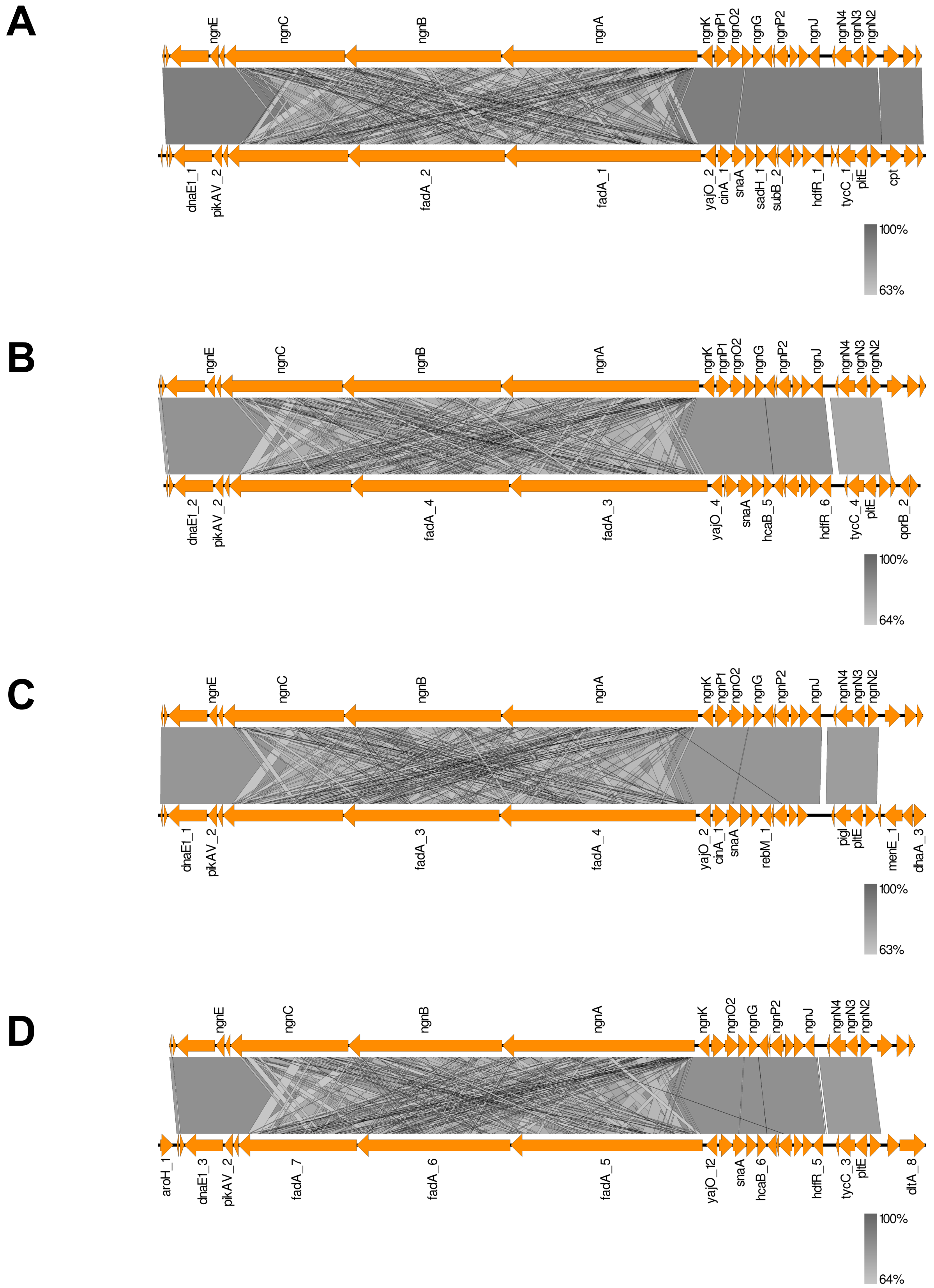


**Supplementary Figure S6.** Sequence alignment of the *ngn* biosynthetic gene cluster. **(A)** Alignment between *Nocardia argentinensis* ATCC 31306 (top) and *Nocardia* sp. CS682 (bottom). **(B)** Alignment between *N. argentinensis* ATCC 31306 (top) and *N. otitidiscaviarum* NEB252 (bottom). **(C)** Alignment between *N. argentinensis* ATCC 31306 (top) and *N. arthritidis* AUSMDU00012717 (bottom). **(D)** Alignment between *N. argentinensis* ATCC 31306 (top) and *N. transvalensis* BJ06-0148 (bottom). Color scale indicates nucleotide sequence similarity.
