# Supplementary material for: Comparative genomics of Nocardia tsunamiensis IFM 10818, a new source of the antibacterial macrolide nargenicin A1
Source: Microbiol Spectr. 2025 Oct 27;13(12):e01220-25. doi: 10.1128/spectrum.01220-25 (PMC12671133; doi:10.1128/spectrum.01220-25)
Supplement: Figure S7 — Phylogenetic relationships based on biosynthetic gene clusters and conservation of gene cluster families. [file spectrum.01220-25-s0007.pdf]

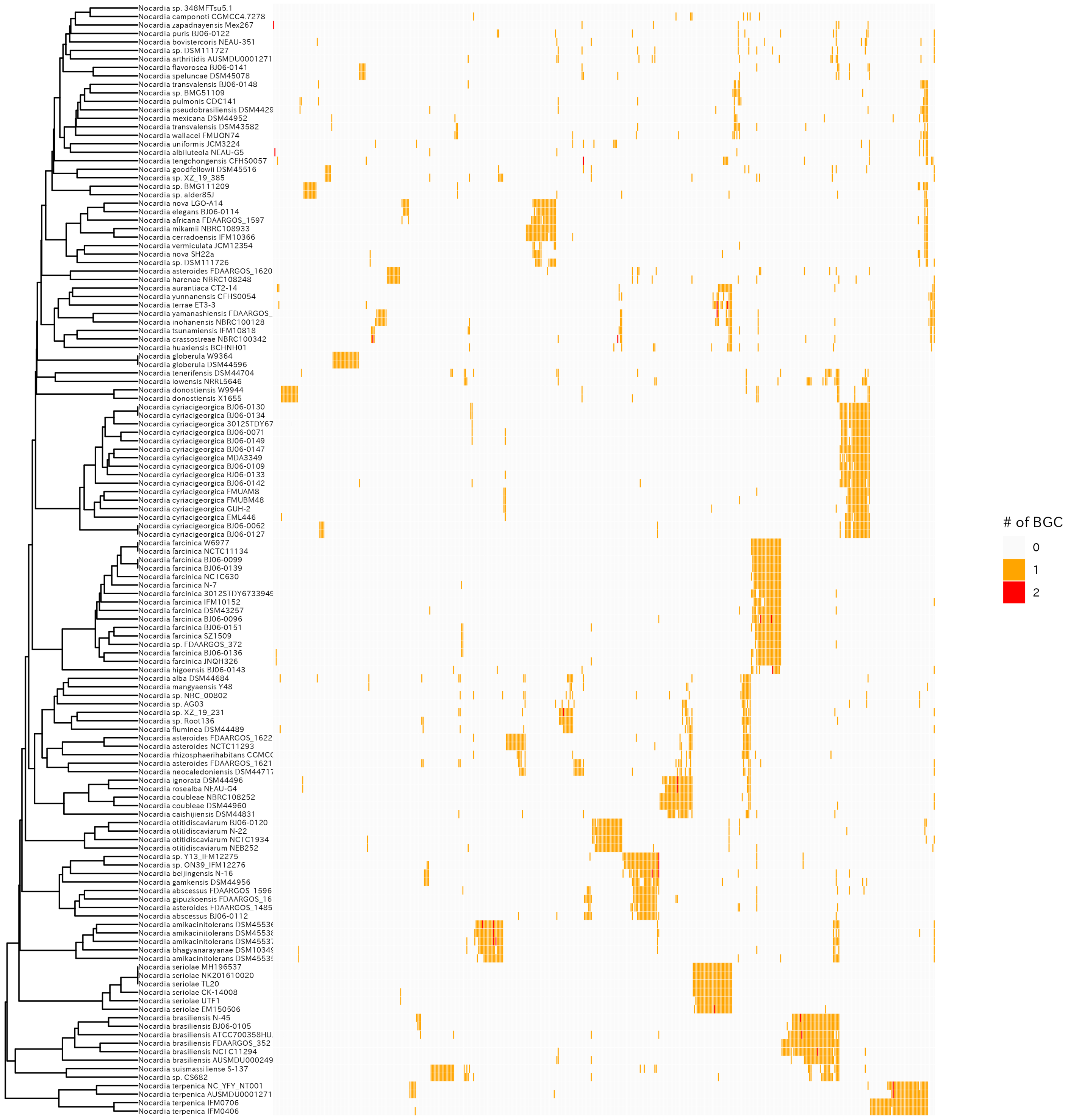

Supplementary Figure S7. Phylogenetic relationships based on biosynthetic gene clusters (BGCs) and conservation of gene cluster families (GCFs). The phylogenetic tree was constructed using the UPGMA method. The presence or absence of 1,456 GCFs is indicated.
