# Supplementary material for: Comparative genomics of Nocardia tsunamiensis IFM 10818, a new source of the antibacterial macrolide nargenicin A1
Source: Microbiol Spectr. 2025 Oct 27;13(12):e01220-25. doi: 10.1128/spectrum.01220-25 (PMC12671133; doi:10.1128/spectrum.01220-25)
Supplement: Figure S8 — Biosynthetic gene clusters present in Nocardia tsunamiensis IFM 10818 and Nocardia crassostreae NBRC 100342. [file spectrum.01220-25-s0008.pdf]

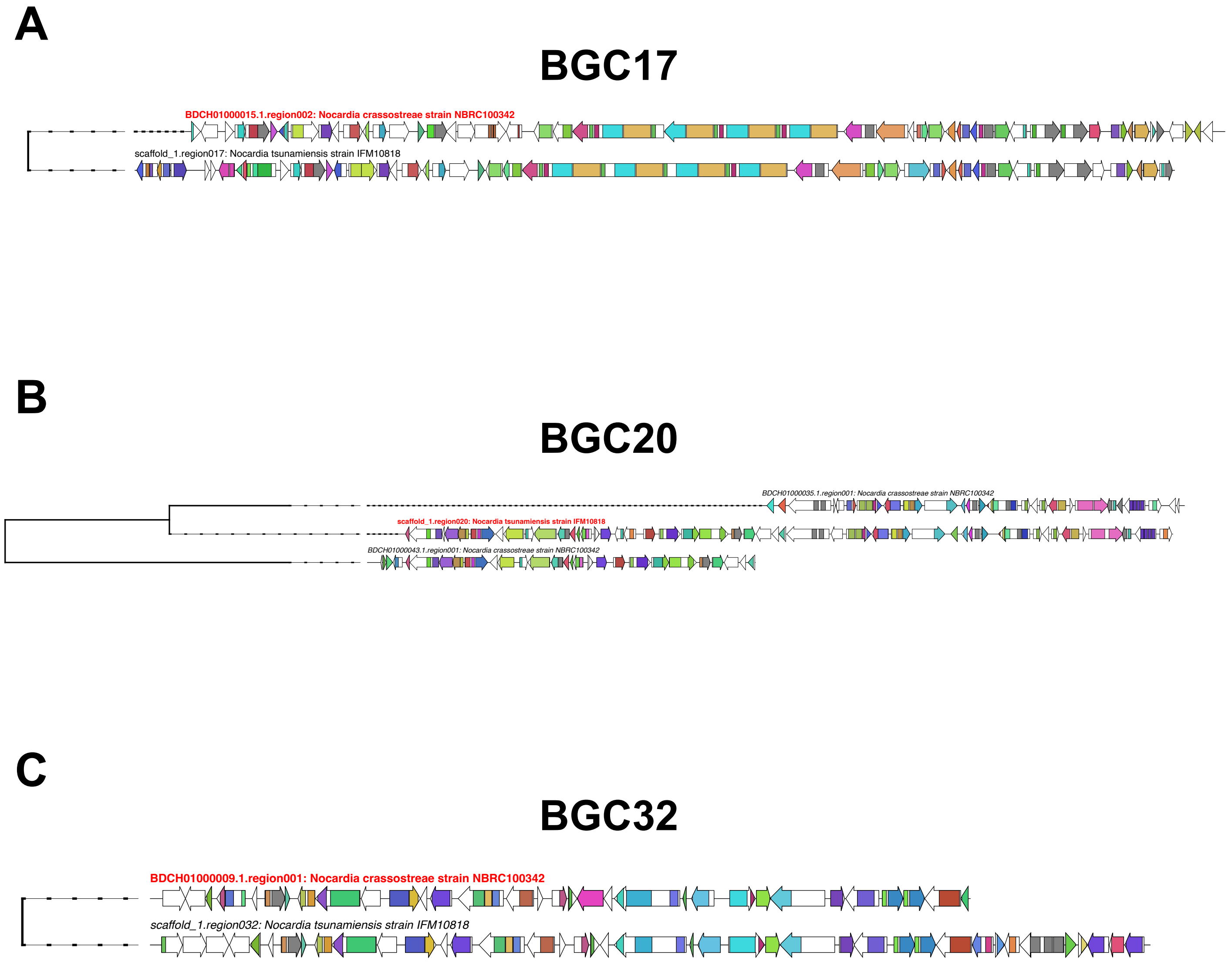

Supplementary Figure S8. Biosynthetic gene clusters (BGCs) present in *Nocardia tsunamiensis* IFM 10818 and *Nocardia crassostreae* NBRC 100342. **(A)** BGC17 (NRPS). **(B)** BGC20 (T3PKS, arylpolyene). Two contigs from the *N. crassostreae* NBRC 100342 genome span this BGC. **(C)** BGC32 (T1PKS).
